# Variability between laboratories performing coagulation tests with identical platforms: a nationwide evaluation study

**DOI:** 10.1186/1477-9560-11-6

**Published:** 2013-03-07

**Authors:** Michael Nagler, Lucas M Bachmann, Lorenzo Alberio, Anne Angelillo-Scherrer, Lars M Asmis, Wolfgang Korte, Adriana Mendez, Guido Reber, Hans Stricker, Dimitrios A Tsakiris, Walter A Wuillemin

**Affiliations:** 1 Division of Hematology and Central Hematology Laboratory, Luzerner Kantonsspital, Lucerne 16 CH-6000, Switzerland; 2 Medignition Inc, Zug CH-6300, Switzerland; 3 Department of Hematology and Central Hematology Laboratory, Inselspital University Hospital and University of Berne, Berne CH-3010, Switzerland; 4 Service and Central Laboratory of Hematology, Centre Hospitalier Universitaire Vaudois and University of Lausanne, Lausanne CH-1011, Switzerland; 5 Unilabs Zurich, Zurich CH-8034, Switzerland; 6 Institute for Clinical Chemistry and Hematology, Cantonal Hospital St Gallen, St Gallen CH-9007, Switzerland; 7 Center for Laboratory Medicine, Cantonal Hospital Aarau, Aarau CH-5001, Switzerland; 8 Laboratory for Special Hemostasis, University Hospital of Geneva, Geneva CH-1211, Switzerland; 9 Division of Surgery, Regional Hospital La Carita, Locarno CH-6600, Switzerland; 10 Diagnostic Hematology, University Hospital of Basel, Basel CH-4031, Switzerland; 11 University of Berne, Berne CH-3000, Switzerland; 12 Division of Hematology, University Hospital and University of Zurich, Zurich CH-8091, Switzerland

**Keywords:** Inter-rater variability, Intraclass correlation coefficient, Reproducibility of testing, Test validity

## Abstract

**Background:**

While the assessment of analytical precision within medical laboratories has received much attention in scientific enquiry, the degree of as well as the sources causing variation between them remains incompletely understood. In this study, we quantified the variance components when performing coagulation tests with identical analytical platforms in different laboratories and computed intraclass correlations coefficients (ICC) for each coagulation test.

**Methods:**

Data from eight laboratories measuring fibrinogen twice in twenty healthy subjects with one out of 3 different platforms and single measurements of prothrombin time (PT), and coagulation factors II, V, VII, VIII, IX, X, XI and XIII were analysed. By platform, the variance components of (i) the subjects, (ii) the laboratory and the technician and (iii) the total variance were obtained for fibrinogen as well as (i) and (iii) for the remaining factors using ANOVA.

**Results:**

The variability for fibrinogen measurements within a laboratory ranged from 0.02 to 0.04, the variability between laboratories ranged from 0.006 to 0.097. The ICC for fibrinogen ranged from 0.37 to 0.66 and from 0.19 to 0.80 for PT between the platforms. For the remaining factors the ICC’s ranged from 0.04 (FII) to 0.93 (FVIII).

**Conclusions:**

Variance components that could be attributed to technicians or laboratory procedures were substantial, led to disappointingly low intraclass correlation coefficients for several factors and were pronounced for some of the platforms. Our findings call for sustained efforts to raise the level of standardization of structures and procedures involved in the quantification of coagulation factors.

## Introduction

While the assessment of analytical precision within one medical laboratory has received much attention in scientific enquiry, extent and sources of variability that occur between laboratories quantifying the same parameter remains incompletely understood. In view of the fact that the clinical value of a laboratory test depends directly on its reproducibility and comparability [1], the scientific community has made great efforts to promote analytical precision within laboratories lately [2-4]. For example, external quality assessment programs (EQA) were introduced to improve comparability between laboratories and are seen as an essential part of quality management systems today [3-5]. Nevertheless, data on variability between laboratories remain limited and analysis of variance components are scarce. Available investigations indicate a large variability, even in the context of coagulation parameter measurements [5-8] thus jeopardizing the comparability of results between different institutions.

On the other hand, knowledge is also scarce for causal sources of variability between laboratories. Possible influences may be the type of the parameter, reagents and calibrators used, the level of standardisation within each laboratory and the adherence to guidelines [9]. If investigations could identify factors that contribute to the variability between laboratories, efforts could be made to improve comparability efficiently. In this study, using the example of haemostasis, we quantified the variance components when performing coagulation tests with identical analytical platforms in different laboratories and computed intraclass correlations coefficients (ICC) for each coagulation test. Thus, we aimed to quantify the extend of variation between state-of-the-art laboratories in relation to the variation of the subjects and to determine the sources of this variation.

## Materials and methods

### Study design and population

This investigation was a secondary analysis based on a previous study of the Swiss RIVAMOS study group [10]. In this multicentre, prospective evaluation study blood samples of 20 eligible and consenting healthy male volunteers were included. The study was approved by the local ethical review board of our institution (Kantonale Ethikkommission Luzern).

### Variance components of coagulation tests between laboratories

Variability of coagulation tests between laboratories is influenced by biological and analytical variability. The two main components of biological variability are intra- and inter-individual variability [11]. Analytical variability between laboratories is influenced (a) by the variability within laboratories, (b) by the platforms used, (c) the assay designs used and (d) variance components that could be attributable to technicians and laboratory procedures (including the choice of reagents and calibrators). The type of coagulation factor measured may also influence the variability. To investigate the variability between laboratories, we included the inter-individual variability, the variability within laboratories, the variability between laboratories and the remaining variability (variance components, that could be attributable to technicians and laboratory procedures) in our statistic model as described below. The remaining factors are considered as stable due to several reasons. The intra-individual variability is regarded as low in coagulation tests and the interval between the two measurements is very short (2–3 hours) [11]. Influence of the platform was addressed as calculations have been done by every platform. The assay designs were identical by platform. Preanalytic factors were considered by applying a strict protocol and using the same technician for serial measurements. Storage and transport of the samples were identical for all laboratories. To avoid influences of different batches of reagents, all determinations were done in one batch in every laboratory.

### Blood withdrawal and handling of the samples

Citrated plasma samples were taken two times in an interval of 2 to 3 hours at the senior author’s institution. Blood withdrawal was done under standardised conditions to preclude preanalytic influences. Plastic syringes (Monovette®, Sarstedt, Nümbrecht, Germany) containing 1 ml trisodium citrate (0.106 mol/l) for 9 ml of blood were used. Citrated samples were collected after EDTA samples to avoid tissue factor contamination of citrated samples. Plasma samples were snap-frozen at – 20°C and shipped under standardised conditions using a commercial courier service with a delivery time of 2–4 hours.

### Laboratories and analysis

All nine haemostasiological laboratories of tertiary hospitals in Switzerland participated in this investigation (In alphabetical order; Cantonal Hospital Aarau, University Hospital of Basel, Inselspital University Hospital of Berne, University Hospital of Geneva, University Hospital of Lausanne, Regional Hospital of Locarno, Cantonal Hospital of Lucerne, Cantonal Hospital of St. Gallen, University Hospital of Zurich). One laboratory was excluded from analysis of between-laboratory variability because of a unique platform. The remaining eight laboratories used one out of three platforms. Details of coagulometers, assays and reagents used are given in Table 1. For determination of variability between laboratories, local protocols were used for testing samples. All laboratories used strict internal and external quality assessment measures, conducted formal test evaluation and are accredited to the Swiss Accreditation Service (SAS). Coagulation tests have been done in accordance with international guidelines [12].

**Table 1:** Participating laboratories with coagulometers, assays and reagents used

**Laboratory**^**¶**^	**Platform**	**Extrinsic coagulation factors**^**‡**^		**Intrinsic coagulation factors**^**§**^		**Fibrinogen**		**Factor XIII**	
		***Assay***	***Reagents***	***Assay***	***Reagents***	***Assay***	***Reagents***	***Assay***	***Reagents***
A ^†^	Instrumentation Laboratory ACL Top 700	Clotting test (photometric)	Recombi-plastin®	Clotting test (photometric)	Hemosil® APPT SP	Clauss method	Hemosil® Fibrinogen-C	Kinetic photometric assay	Berichrom® FXIII
B	Siemens BCS-XP	Clotting test (photometric)	Innovin®	Clotting test (photometric)	Actin®	Clauss method	Multifibren U	Kinetic photometric assay	Berichrom® FXIII
C	Roche STA-R	Clotting test (viscosity)	Innovin®	Clotting test (viscosity)	Actin FS®	Clauss method	STA® Fibrinogen	Latex immunturbidimetry	HEXAMATE® FXIII
D	Sysmex CA7000	Clotting test (photometric)	Innovin®	Clotting test (photometric)	Actin®	Clauss method	Dade® Thrombin	n.a.	n.a.
E	Siemens BCS-XP	Clotting test (photometric)	Innovin®	Clotting test (photometric)	Pathromtin® SL	Clauss method	Multifibren U	Kinetic photometric assay	Berichrom® FXIII
F	Siemens BCS	Clotting test (photometric)	Innovin®	Clotting test (photometric)	Actin FS®	Clauss method	Multifibren U	Kinetic photometric assay	Berichrom® FXIII
G^*^	Sysmex CA1500	Clotting test (photometric)	Innovin®	Clotting test (photometric)	Actin FS®	Clauss method	Dade® Thrombin	n.a.	n.a.
H	Siemens BCS-XP	Clotting test (photometric)	Innovin®	Clotting test (photometric)	Pathromtin® SL	Clauss method	Multifibren U	Kinetic photometric assay	Berichrom® FXIII
I	Roche STA-R	Clotting test (viscosity)	Innovin®	Clotting test (viscosity)	STA® APPT	Clauss method	bovine thrombin, Diagnotec AG	Kinetic photometric assay	Berichrom® FXIII

^¶^ Random order, not matching with the alphabetical list mentioned above. ^*^ Laboratory was excluded from analysis of variability between laboratories because not all tests were available. ^†^ Laboratory was excluded from analysis of variability between laboratories because of a unique platform. ^‡^ PT, Factors II, V, VII, X. ^§^ Factors VIII, IX, XI.

Fibrinogen was determined in plasma samples of both points in time. Prothrombin time (PT), factors II, V, VII, VIII, IX, X, XI and XIII were determined only for the first sample to avoid possible interferences with the second investigation conducted [10].

### Statistical analysis

To study the variability between the different laboratories, we performed analyses of variance (ANOVAs) and used the variance components procedure. By platform, the variance components of (i) the subjects, (ii) the laboratory and the technician (including the measurement error of the platform) and (iii) the total variance were obtained for fibrinogen as well as (i) and (iii) for the remaining factors.

Based on the variance components, we calculated the intraclass correlation coefficients (ICC) for all factors using the variance component of the subjects in the numerator and the total variance in the denominator. Thus, the variation of the measurements between laboratories can be demonstrated in relation to the variation of the subjects (what represents the ‘real’ variation). A high ICC (clearly above 0.5) refers to the subjects as main components of the variance (which is a desirable outcome) whereas a low ICC indicates that other (non-subject) factors are more accountable (which is a disturbing outcome).

Statistical analysis was performed using the SPSS statistical software package (Version 19; Property of SPSS Inc an IBM Company. © Copyright SPSS Inc. 1989, 2010).

## Results

A total of 360 fibrinogen measurements, 180 PT measurements, 160 F II, F V, F VII, F VIII, F IX, F X and F XI measurements, and 140 F XIII measurements were available for analysis because only fibrinogen was determined two times and one laboratory did not provided all tests. Raw data for all analysis are available at Additional file 1: Table S1.

The intraobserver variability for fibrinogen measurements, indicating the variability within a laboratory, ranged from 0.02 to 0.04. This variability occurs from the variability within a laboratory (including the technician handling the samples). The variability between laboratories ranged from 0.006 to 0.097. This indicates that laboratories used the platform in different ways or that they interpreted the manual differently. This detailed analysis was only possible for the fibrinogen measurement because fibrinogen was determined in plasma samples of both points in time.

The results allow discussing how prone a platform – the specifications, the complexity in the handling and other reasons – might be to create higher variability when used in different laboratories and contexts.

The ICC for fibrinogen ranged from 0.37 to 0.66 and from 0.19 to 0.80 for PT between the laboratories. Though, the amount to which non-subject factors is accountable for the variance of the parameters is very varying. Low ICC indicates that non-subject factors are major components of variance.

The ICC of FII when assessed with platform 3 was as low as 0.04 as compared to 0.44 when measured with platform 2. For the remaining factors the ICC’s ranged from 0.22 (F V) assessed with platform 3, to 0.93 (F VIII) assessed with platform 2.

For the two platforms providing enough data, the average ICC across all parameters was 0.64 (standard deviation 0.25) for platform 2 and 0.56 (standard deviation 0.28) for platform 3. Overall, the ICC’s were at 0.60 on average. Details are available on Table 2.

**Table 2:** Intraclass correlations of various coagulation tests and intraobserver variability of fibrinogen tests with various platforms

**Parameter**	Platform 1*	**Platform 2**	**Platform 3**	**Platform 4**^**‡**^
**# laboratories**	1	2	4	2
**Fibrinogen**	0.85	0.37	0.65	0.66
Within- laboratory variability ¶		0.02	0.03	0.04
Between- laboratory variability +		0.097	0.03	0.006
**Prothrombin time**		0.19	0.24	0.80
**F II**		0.44	0.04	
**F V**		0.63	0.22	
**F VII**		0.71	0.82	
**F VIII**		0.93	0.61	
**F IX**		0.86	0.83	
**F X**		0.82	0.70	
**F XI**		0.90	0.65	
**F XIII**		0.57	0.81	

* laboratory was excluded from analysis of between-laboratory variability because of a unique platform, ‡ analysis was limited to routine coagulation parameters, because one laboratory did not assess all tests, ¶ (Platform + Technician), + Laboratories use the platform in different ways (they interpret the manual differently).

## Discussions

Variance components that could be attributed to technicians or laboratory procedures were substantial, led to disappointingly low intraclass correlation coefficients for several factors and were pronounced for some of the platforms, probably for those allowing more instrument adjustments. Our results emphasise that variability of parameters between state-of-the-art laboratories remains considerable. This may have a relevant impact on the reproducibility and comparability of the results.

Our results confirm and extend previous reports on a wide variability of coagulation parameters. A wide variability of PT measurements was already noticed with the introduction of early automation coagulometers [13]. An investigation of the World Federation of Haemophilia (WFH) EQA programme and the United Kingdom National External Quality Assessment Scheme (UK NEQAS) conducted in the UK and in emerging countries revealed a good to acceptable variation with regard to PT and aPTT (coefficient of variation [CV]: 10.1-20.4%) but an extensive variation with regard to FVIII, FIX and von Willebrand factor determination (CV 6-154%) [14]. Considerable variations in determination of von Willebrand factor antigen (vWF:Ag) and ristocetin cofactor activity (vWF:RCo) were recognised in investigations of the U.S. College of American Pathologists proficiency testing (CV 3.2-30.9%; n = 171 laboratories) [14], the European Concerted Action on Thrombosis and Disabilities Foundation (CV 10-40%; n = 181 laboratories) [15] and UK NEQAS (CV 15-50%; n = 200) [16].

Agreement with regard to subtherapeutic, therapeutic or supratherapeutic levels in the monitoring of unfractionated heparin using activated partial thromboplastin time or anti-Xa level was very limited in a cross-validation study [17] as well as a study using the results of the annual Ontario Quality Management Program [6]. An interlaboratory agreement of 16-52% and a coefficient of variation of 10.5 – 65% were reported respectively. Another investigation used the results of the Italian External Quality Assessment Scheme and found a wide variability in estimation of quantitative D-Dimers (coefficient of variation up to 47%) [18]. A high degree of variation between laboratories in the identification of coagulation factor inhibitors was shown in several investigations, which used data of external quality assessment programs [7,19-21]. Other investigations identified the type of the reagent [22], the method of the assay [23] and the calibrator [24,25] as factors that bias the measurements between different laboratories and the coagulometer influencing the precision of the measurements [22].

In contrast to these previous investigations, our study illustrates the degree of variation between laboratories using state-of-the-art coagulometers and reagents in relation to the variation of the subjects. We showed the degree of variation for PT, fibrinogen and coagulation factors II, V, VII, VIII, IX, XI and XIII. Furthermore, we characterised factors associated with technicians and laboratory procedures as a relevant source of this variation. These factors include also the choice of the reagents, which may have influenced the higher between-laboratory variability of platform 2 in contrast to platform 3.

Our investigation has several limitations. First, it is an exploratory study with a relative low number of determinations. However, it facilitates future investigations with more determinations and a proper power analysis. Second, “factors associated with technicians and laboratory procedures” are determined as a global factor. The design of the study did not allow discriminating additional potential factors such as type of the reagents, which are also inadequately addressed by the literature.

Future investigations have to separate these factors to discriminate between organisational factors and single technicians. Third, only fibrinogen determination was analysed for two points in time, because of the study design. If future investigations would determine more parameters two times, better information on differences between parameters would be possible. Forth, only state-of-the-art laboratories specialised in determination of coagulation factors measured the samples in our investigation. The results could possibly be different if routine laboratories would have been investigated.

The strength of our study is that was done it in a nation-wide design including only state-of-the-art coagulation laboratories of all tertiary hospitals. Therefore, a selection bias appears unlikely. Furthermore, a wide range of coagulation parameters was determined. Though, the observed effects were internally confirmed by other parameters. Moreover, it is one of the first investigations that observed the sources of this variation and – to our knowledge – the first investigation, which observed factors associated with technicians and laboratory procedures as possible source for variation of results between laboratories.

The present results suggest that variability of coagulation parameters between specialised laboratories is substantial despite state-of-the-art coagulometers and reagents, great efforts to guarantee precision of laboratory tests and external quality assessment programs. The results indicate that factors associated with technicians and laboratory procedures are a relevant source of this variation. Though, organisational factors are a promising field of work for future investigations on the sources of this variability. Furthermore, efforts to enhance laboratory organisation may be an attractive area of activity for reducing variability of the measurements of coagulation parameters as well as improving quality of laboratory results within laboratories. This hypothesis is supported by the fact that platforms, which allow more adjustments, were associated with a greater variance than others with a more rigid application. Furthermore, parameters, which necessitate a greater effort in structuring operation procedure, have had a lower variance than more simple parameters.

In conclusion, variability between laboratories in determination of coagulation parameters was considerable and variance components that could be attributable to technicians and laboratory procedures were substantial. Once confirmed in larger studies, our findings call for sustained efforts to raise the level of standardization of structures and procedures involved in the quantification of coagulation factors.

## Abbreviations

EQA: External quality assessment program; ICC: Intraclass correlation coefficient; ANOVA: Analysis of variance.

## Competing interests

The authors declare that they have no competing interests.

## Authors’ contributions

LA, AA-S, LMA, WK, AM, GR, HS, DAT and WAW designed the RIVAMOS study, performed the research and collected the data. MN, LMB and WAW designed the present part of the investigation, analysed the data and drafted the manuscript. All authors revised the manuscript, added important intellectual content and approved the final version of the manuscript.

Part of the investigation was presented at the 56th meeting of the “Gesellschaft für Thrombose und Hämostaseforschung (GTH)” in St. Gallen, Switzerland, February 1–4, 2012.

## Supplementary Material

Additional file 1: Table S1 Raw data of coagulation tests obtained by platform. Click here for file
