# Supplementary material for: Variability between laboratories performing coagulation tests with identical platforms: a nationwide evaluation study
Source: Thromb J. 2013 Mar 7;11:6. doi: 10.1186/1477-9560-11-6 (PMC3599351; doi:10.1186/1477-9560-11-6)
Supplement: Additional file 1: Table S1 — Raw data of coagulation tests obtained by platform. [file 1477-9560-11-6-S1.docx]

Additional file 1: Table S1. Raw data of coagulation tests obtained by platform

| **Parameter** | **Distribution**  *n; mean (SD)* | | | |
| --- | --- | --- | --- | --- |
|  | **Platform 1** | **Platform 2** | **Platform 3** | **Platform 4** |
| **Prothrombin time**  (n=180;% Quick) | 20; 96.9 (± 7.3) | 40; 88.3 (± 8.1) | 80; 95.8 (± 11.5) | 40; 86.3 (± 6.1) |
| **Fibrinogen t1**  (n=180; g/l) | 20; 2.4 (± 0.3) | 40; 2.5 (± 0.4) | 80; 2.8 (± 0.4) | 40; 2.3 (± 0.4) |
| **Fibrinogen t2**  (n=180; g/l) | 20; 2.4 (± 0.3) | 40; 2.4 (± 0.4) | 80; 2.7 (± 0.4) | 40; 2.2 (± 0.4) |
| **Factor II**  (n=160; %) | 20; 100.3 (± 8.0) | 40; 97.2 (± 9.8) | 80; 112.4 (± 15.1) | 20; 100.6 (± 18.2) |
| **Factor V**  (n=160; %) | 20; 109.2 (± 13.7) | 40; 104.2 (± 15.6) | 80; 114.7 (± 19.0) | 20; 117.1 (± 28.3) |
| **Factor VII**  (n=160; %) | 20; 77.6 (± 18.7) | 40; 90.1 (± 16.8) | 80; 96.2 (± 18.0) | 20; 97.8 (± 21.9) |
| **Factor X**  (n=160; %) | 20; 98.5 (± 13.4) | 40; 88.2 (± 11.8) | 80; 105.6 (± 15.0) | 20; 98.4 (± 21.8) |
| **Factor VIII**  (n=160; %) | 20; 93.6 (± 20.1) | 40; 94.7 (± 25.7) | 80; 90.5 (± 22.5) | 20; 103.0 (± 30.0) |
| **Factor IX**  (n=160; %) | 20; 108.6 (± 16.6) | 40; 100.9 (± 17.4) | 80; 95.4 (± 13.6) | 20; 98.9 (± 19.8) |
| **Factor XI**  (n=160; %) | 20; 117.0 (± 16.3) | 40; 103.0 (± 15.1) | 80; 107.6 (± 15.5) | 20; 108.3 (± 20.5) |
| **Factor XIII**  (n=140; %) | 20; 108.7 (± 16.1) | 40; 109.4 (± 24.3) | 80; 109.8 (± 22.9) | n.a. |
